# Usefulness of PET/CT for early detection of internal malignancies in patients with Muir–Torre syndrome: report of two cases

**DOI:** 10.1186/s40792-017-0346-7

**Published:** 2017-05-23

**Authors:** Yui Ishiguro, Shigenori Homma, Tadashi Yoshida, Yosuke Ohno, Nobuki Ichikawa, Hideki Kawamura, Hiroo Hata, Satoru Kase, Susumu Ishida, Hiromi Okada-Kanno, Kanako C. Hatanaka, Akinobu Taketomi

**Affiliations:** 1 Department of Gastroenterological Surgery I, Hokkaido University Graduate School of Medicine, North 15, West 7, Kita-Ku, Sapporo, Hokkaido 060-8638 Japan; 2 Department of Dermatology, Hokkaido University Graduate School of Medicine, North 15, West 7, Kita-Ku, Sapporo, Hokkaido 060-8638 Japan; 3 Department of Ophthalmology, Hokkaido University Graduate School of Medicine, North 15, West 7, Kita-Ku, Sapporo, Hokkaido 060-8638 Japan; 4 Department of Surgical Pathology, Hokkaido University Hospital, North 14, West 5, Kita-Ku, Sapporo, Hokkaido 060-8648 Japan

**Keywords:** Muir–Torre syndrome, PET/CT, Sebaceous carcinoma, Internal malignancy

## Abstract

**Background:**

Muir–Torre syndrome (MTS) is a rare autosomal dominant genodermatosis caused by mutations in mismatch repair genes. It is characterized by the presence of at least one sebaceous skin tumor associated with internal malignancies. Whether positron emission tomography/computed tomography (PET/CT) is useful for the detection of malignancies in patients with MTS has not been determined. We herein report two cases in which PET/CT was useful for the diagnosis and follow-up of internal malignancies in patients with MTS.

**Case presentation:**

In case 1, a 57-year-old woman underwent excision of a sebaceous carcinoma on the left upper eyelid. She underwent follow-up PET/CT once yearly thereafter. Forty-two months after the eyelid surgery, PET/CT showed intense tracer uptake in the right lower abdomen. An ascending colon tumor was identified, and examination of a biopsy specimen showed adenocarcinoma. In case 2, a 77-year-old man presented for evaluation of three continuous papules with telangiectasia on his right cheek. Examination of a skin biopsy specimen of the cheek papule revealed a sebaceous carcinoma. He underwent PET/CT to detect other malignancies. PET/CT showed intense tracer uptake in the sigmoid colon. A sigmoid colon tumor was identified, and examination of a biopsy specimen showed adenocarcinoma. Both patients underwent resection of their tumors, and both were still free of recurrence of the sebaceous and colon carcinomas at the time of this writing.

**Conclusion:**

PET/CT is a reliable imaging modality for the detection of internal malignancies and is useful for the diagnosis and follow-up of MTS.

## Background

Muir–Torre syndrome (MTS) is a rare autosomal dominant genodermatosis characterized by the presence of both sebaceous skin tumors and internal malignancies; it is also considered a subtype of hereditary nonpolyposis colorectal cancer [1]. MTS was first reported by Muir et al. in 1967 [2] and Torre in 1968 [3]. The underlying genetic background of MTS is attributed to microsatellite instabilities caused by germline mutations in DNA mismatch repair genes, most commonly *MSH2* and *MLH1* and less commonly *MSH6*, which are responsible for base substitution mutations [4, 5].

MTS is associated with the development of many kinds of internal malignancies. Although Ponti and Ponz de Leon [1] suggested the necessity of surveillance for MTS gene carriers and symptomatic patients with MTS, the effectiveness of such surveillance has been controversial because of the difficulty of screening for various kinds of malignancies.

We herein report two cases of MTS in which positron emission tomography/computed tomography (PET/CT) was useful in the diagnosis and surveillance of internal malignancies.

## Case presentation

### Case 1

**Fig. 1:**
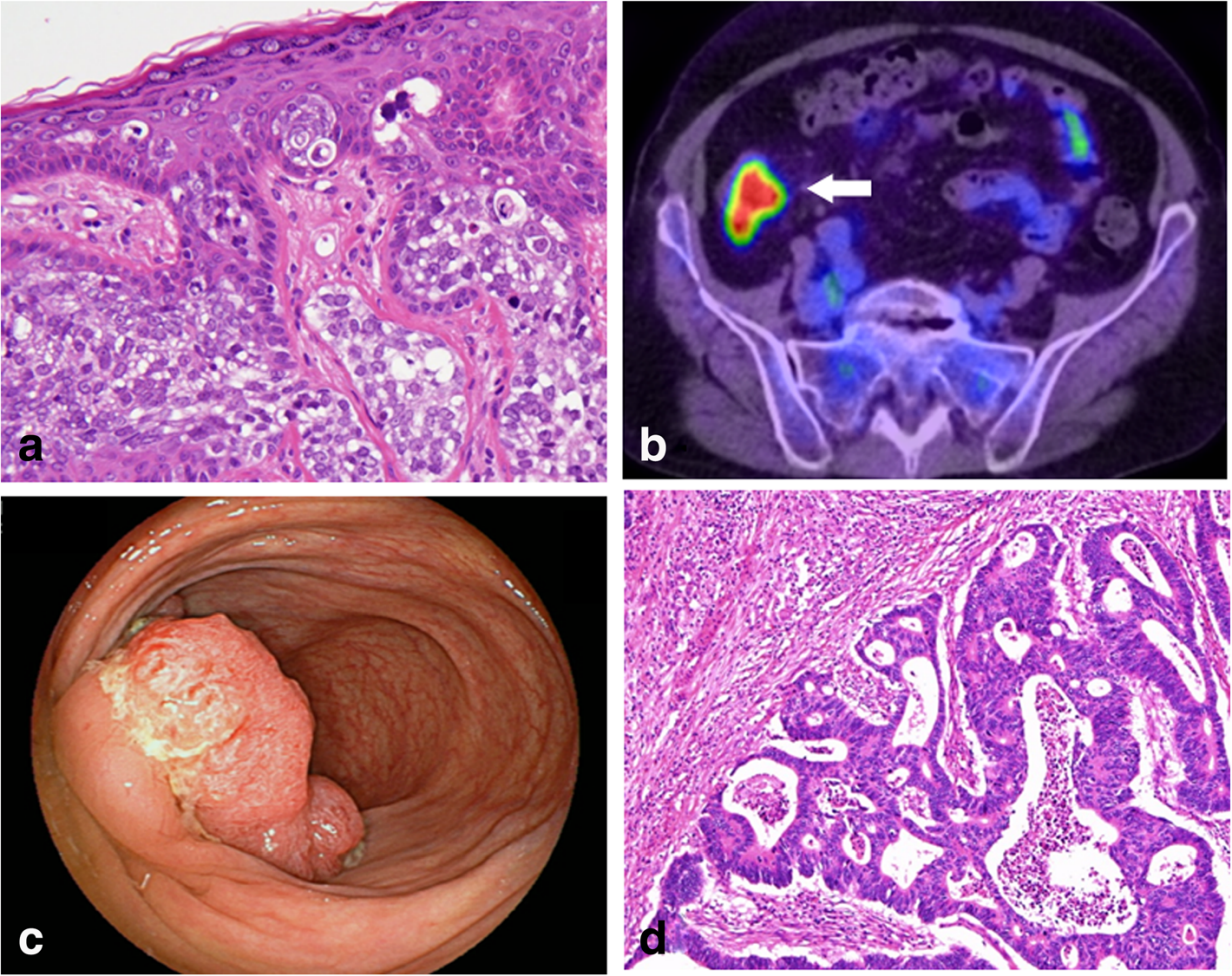
Clinicopathological findings of case 1. **a** Pathological examination of a 5-mm-diameter skin tumor on the left upper eyelid revealed sebaceous carcinoma (hematoxylin and eosin stain (H.E.), ×200). **b** Positron emission tomography/computed tomography showed intense tracer uptake (maximum standardized uptake value = 16.8) in the right lower abdomen (*white arrow*). **c** Colonoscopy showed a type 2 tumor in the ascending colon. **d** Pathological examination of the ascending colon tumor revealed heterogeneity of moderately and well-differentiated adenocarcinoma (H.E., ×100)

A 57-year-old woman underwent excision of a 5-mm-diameter skin tumor on the left upper eyelid in April 2010. Histopathological examination of the tumor showed a sebaceous carcinoma (Fig. 1a). The patient underwent follow-up PET/CT once yearly thereafter. Forty-two months after the eyelid surgery, PET/CT showed intense tracer uptake (maximum standardized uptake value = 16.8) in the right lower abdomen (Fig. 1b). Her family’s medical history was negative for internal malignancy. Concentrations of the tumor markers carcinoembryonic antigen (CEA) and cancer antigen 19-9 were 6.2 ng/mL and 624.4 U/mL, respectively. Colonoscopy showed a type 2 tumor in the ascending colon (Fig. 1c), and examination of a biopsy specimen showed adenocarcinoma. She underwent laparoscopic ileocecal resection. Histopathological examination of the surgical specimen showed a 39 × 32 mm type 2 tumor in the cecum exhibiting heterogeneity of moderately and well-differentiated adenocarcinoma (Fig. 1d). Furthermore, seven lymph node metastases were detected (T2 N2b M0 stage IIIB according to the 7th edition of the UICC classification). Although MTS was suspected, the patient refused genetic analysis. Considering the pathological findings of the colon carcinoma, she received adjuvant chemotherapy with capecitabine and oxaliplatin for 6 months. She was free of recurrence of the sebaceous and colon carcinomas at the time of this writing (72 months after the first surgery).

### Case 2

**Fig. 2:**
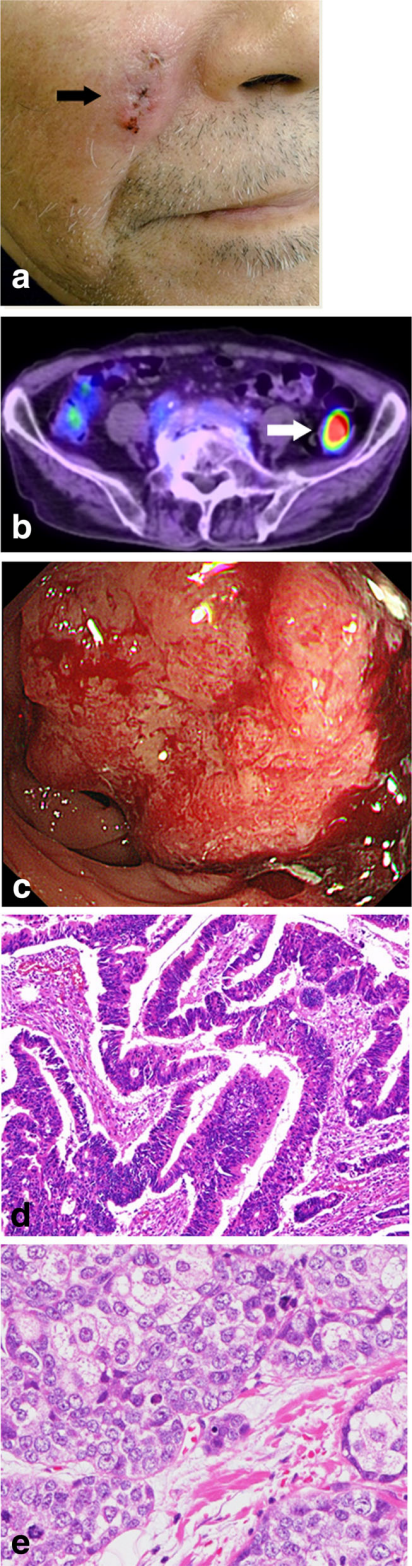
Clinicopathological findings of case 2. **a** Continuous papules measuring 15 × 11 mm with telangiectasia were present on the right side of the cheek (*black arrow*). **b** Positron emission tomography/computed tomography showed intense tracer uptake (maximum standardized uptake value = 36.8) at the sigmoid colon (*white arrow*). **c** Colonoscopy showed an ulcerated hemorrhagic type 2 tumor at the sigmoid colon. **d** Pathological examination of the sigmoid colon tumor revealed heterogeneity of well-differentiated adenocarcinoma, moderately differentiated adenocarcinoma, and mucinous adenocarcinoma (hematoxylin and eosin stain (H.E.), ×100). **e** Pathological examination of the tumor on the right cheek revealed sebaceous carcinoma (H.E., ×200)

A 77-year-old man with a medical history of diabetes and tuberculosis presented for evaluation of three continuous papules with telangiectasia on his right cheek. His family’s medical history included gastric carcinoma in his father and brother. The papules featured a 15 × 11 mm non-tender induration (Fig. 2a), and examination of a skin biopsy specimen revealed a sebaceous carcinoma. The tumor marker levels were within their reference ranges (CEA, 4.7 ng/mL; cancer antigen 19-9, 15.1 U/mL). The patient underwent PET/CT to detect other malignancies. PET/CT showed intense tracer uptake (maximum standardized uptake value = 36.8) in the sigmoid colon (Fig. 2b). Colonoscopy showed an ulcerated hemorrhagic type 2 tumor in the sigmoid colon (Fig. 2c). Examination of a biopsy specimen of the sigmoid colon tumor showed adenocarcinoma. First, the patient underwent laparoscopic sigmoidectomy. Histopathological examination of the sigmoid colon showed a 35 × 30 mm type 2 tumor exhibiting heterogeneity of well-differentiated adenocarcinoma, moderately differentiated adenocarcinoma, and mucinous adenocarcinoma (Fig. 2d). Furthermore, two lymph node metastases were detected (T3 N1b M0 stage IIIB). Genetic analyses of the colon cancer revealed two *MSH2* gene mutations: a heterozygous missense mutation from CAG to AAG in codon 419 on exon 7 and a missense mutation from CAA to CGA in codon 629 on exon 12. The patient was therefore diagnosed with MTS and underwent hereditary counseling. One month after the sigmoidectomy, he underwent excision of the sebaceous carcinoma and radical dissection of the right submandibular region. Histopathological examination of the skin tumor revealed a 21 × 20 × 15 mm sebaceous adenoma with free margins (Fig. 2e). Considering the patient’s poor performance status, he did not undergo adjuvant chemotherapy. Although he did not develop recurrence of the sebaceous or colon carcinoma for 24 months after the first surgery, he was lost to follow-up thereafter.

### Discussion

In this study, we demonstrated that a continuous whole-body search with PET/CT was useful in the diagnosis and follow-up of MTS. Patients with MTS develop internal malignancies in various organs and/or at various times. Coldron and Reid [6] reported that nearly half of patients with MTS had two or more internal malignant diseases. Among them, 10% of patients with MTS had more than four internal malignancies, and surprisingly, one patient had nine internal malignancies. Akhtar et al. [7] reported a variety of locations of primary internal malignancies in 399 patients with MTS: the colorectum (56%), urogenital system (22%), breast (4%), small intestine (4%), and head/neck (3%). The age at the time of diagnosis of the initial internal malignancy reportedly ranges from 23 to 81 years, with a median of 50 years [8]. The internal malignancy may appear either synchronously with or metachronously to the sebaceous tumor. In the abovementioned study of 205 patients, the proportions of patients with onset of their sebaceous tumor before, coincident with, and after their internal malignancy were 22, 6, and 56%, respectively [7]. The sebaceous carcinomas in cases 1 and 2 of the present study appeared before and coincident with the internal malignancy, respectively. In most patients with MTS, internal malignancies have surprisingly indolent courses with long patient survival even after the development of metastasis [8, 9]. Thus, patients with MTS should be followed up for a long period of time.

Ponti and Ponz de Leon [1] suggested a follow-up program for gene carriers and symptomatic patients with MTS involving measurement of the CEA concentration, chest radiography, mammography, upper gastrointestinal endoscopy, CT of the abdomen and pelvis, colonoscopy or barium enemas, urine cytology, cervical smears, and endometrial biopsy. This follow-up program is certainly useful for cancer detection, but the effectiveness of these frequent screenings seems questionable in terms of the cost–benefit ratio and stress experienced by patients with MTS. We consider that PET/CT is a suitable screening method that can be used to detect a wide range of malignancies at one time with minimal invasiveness for patients with MTS. Indeed, we performed PET/CT annually and detected a colon carcinoma in case 1. Based on our cases, we propose that patients with MTS or suspected MTS should undergo PET/CT at the time of diagnosis and at the annual follow-up. If patients are symptomatic, other complete examinations such as upper gastrointestinal endoscopy, colonoscopy, and endometrial biopsy should be performed.

A major concern of cancer screening using PET/CT is the balance between the disadvantages and advantages of this technique. Limitations of PET/CT include the fact that some malignancies are difficult to discover by screening PET/CT. It is particularly difficult to detect malignancies in organs that exhibit substantial physiological accumulation such as the brain, renal pelvis, and bladder [10]. However, PET/CT has a high sensitivity (100%) and specificity (43%) for detection of colorectal carcinoma and seems to be superior to CT [11]. Baek et al. [12] described five cases of periocular sebaceous carcinoma in which PET/CT correctly identified four of four cases of regional lymph node involvement, whereas three of these four cases were undetected with CT alone. PET/CT seems to have potential to detect a wide variety of cancers in asymptomatic individuals. Because MTS is a rare disease, very few reports on the utility of PET/CT in patients with MTS are available. Further studies are needed to confirm the effectiveness of PET/CT for cancer screening in patients with MTS.

## Conclusions

In summary, we have herein described two patients with MTS whose colon carcinomas were detected by screening PET/CT. PET/CT seems to be a useful method for the diagnosis and follow-up of MTS.

## Abbreviations

CEA: Carcinoembryonic antigen
MTS: Muir–Torre syndrome
PET/CT: Positron emission tomography/computed tomography
